# Conspecific density dependence and community structure: Insights from 11 years of monitoring in an old‐growth temperate forest in Northeast China

**DOI:** 10.1002/ece3.3050

**Published:** 2017-06-07

**Authors:** Xu Kuang, Kai Zhu, Zuoqiang Yuan, Fei Lin, Ji Ye, Xugao Wang, Yunyun Wang, Zhanqing Hao

**Affiliations:** ^1^ CAS Key Laboratory of Forest Ecology and Management Institute of Applied Ecology Chinese Academy of Sciences Shenyang China; ^2^ University of Chinese Academy of Sciences Beijing China; ^3^ Department of BioSciences Rice University Houston TX USA; ^4^ Department of Biology University of Texas Arlington TX USA

**Keywords:** Changbaishan (CBS) plot, Janzen–Connell hypothesis, long‐term monitoring, population dynamics, spatial pattern analyzes

## Abstract

Forest community structure may be influenced by seedling density dependence, however, the effect is loosely coupled with population dynamics and diversity in the short term. In the long term the strength of conspecific density dependence may fluctuate over time because of seedling abundance, yet few long‐term studies exist. Based on 11 years of seedling census data and tree census data from a 25‐ha temperate forest plot in Northeast China, we used generalized linear mixed models to test the relative effects of local neighborhood density and abiotic factors on seedling density and seedling survival. Spatial point pattern analysis was used to determine if spatial patterns of saplings and juveniles, in relation to conspecific adults, were in accordance with patterns uncovered by conspecific negative density dependence at the seedling stage. Our long‐term results showed that seedling density was mainly positively affected by conspecific density, suggesting dispersal limitation of seedling development. The probability of seedling survival significantly decreased over 1 year with increasing conspecific density, indicating conspecific negative density dependence in seedling establishment. Although there was variation in conspecific negative density dependence at the seedling stage among species and across years, a dispersed pattern of conspecific saplings relative to conspecific adults at the local scale (<10 m) was observed in four of the 11 species examined. Overall, sapling spatial patterns were consistent with the impacts of conspecific density on seedling dynamics, which suggests that conspecific negative density dependence is persistent over the long term. From the long‐term perspective, conspecific density dependence is an important driver of species coexistence in temperate forests.

## 1 INTRODUCTION

Conspecific negative density dependence (CNDD) has long been recognized as a mechanism that maintains species diversity (Antonovics & Levin, 1980; Connell, 1971; Harms, Wright, Calderón, Hernández, & Herre, 2000; HilleRislambers, Adler, Harpole, Levine, & Mayfield, 2012; Janzen, 1970; Wills, Condit, Foster, & Hubbell, 1997). CNDD posits that specialist natural enemies keep the density of each species in check through impacting propagule (seed and/or seedling) survival, which provides space near adult trees for other species, thereby promoting species coexistence and maintaining diversity (Blundell & Peart, 2004; Clark & Clark, 1989; Swamy et al., 2011). CNDD emphasizes the effects of conspecific neighbors on propagule survival, which is prevalent at the seedling stage (Clark & Clark, 1989; Comita & Hubbell, 2009; Gripenberg et al., 2014). In addition to conspecific neighbors, the seedling stage which reflects propagule success, is vulnerable to other factors, such as heterospecific neighbors and abiotic factors (Paine et al., 2011; Queenborough, Burslem, Garwood, & Valencia, 2009; Shibata et al., 2010; Yan, Zhang, Wang, Zhao, & von Gadow, 2015). Seedling survival shows a positive correlation with increasing local heterospecific density, which has been shown to offset the effects of conspecific density (Comita, Muller‐Landau, Aguilar, & Hubbell, 2010). Similarly, a species will tend to perform better in an ideal habitat (Getzin, Wiegand, Wiegand, & He, 2008; Murrell, 2009). When a species has a high local density in marginal habitats or low local density in preferred habitats, conspecific dependence may show similar effects as habitat advantages. So heterospecific neighbors and abiotic factors should be taken into account when attempting to examine conspecific density dependence.

The relation between the strength of CNDD and the relative abundance of host tree species is subject to debate for rare species (Johnson, Beaulieu, Bever, & Clay, 2012; Klironomos, 2002; Mangan et al., 2010) and common species (Bagchi et al., 2014; Kobe & Vriesendorp, 2011; Webb & Peart, 2000; Zhu, Woodall, Monteiro, & Clark, 2015). Moreover, rare species may be rare because they are subject to stronger CNDD than common species when rare species have equal density of conspecific neighbors as common species (Comita et al., 2010; Hubbell, Ahumada, Condit, & Foster, 2001). The key to CNDD is that specialist natural enemies are maintained by conspecific neighbors. A high conspecific density has a strong negative effect on seedlings, while a sufficiently low conspecific density might have very little effect because some natural enemies may disappear (Liu, Fang, Chesson, & He, 2015). Thus, if there is no conspecific density gradient, it is difficult to identify significant effects of CNDD for both common and rare species. The differing effects of CNDD between common species and rare species are also related to seedling abundance itself. Seedlings with low abundance rarely cooccur with conspecific neighbors and are seldom distributed uniformly in different conspecific density gradients (Zhu, Woodall, et al., 2015). Not only do numerous rare species tend to have low seedling abundances, but most common species also do, especially in temperate forest ecosystems. Few studies have focused on the relationships between CNDD variation and seedling abundance.

Most studies about the effect of CNDD on seedlings have been short term experiments (Bai et al., 2012; Chen et al., 2010; Johnson et al., 2014; Lu et al., 2015). The effect of CNDD is often limited to the duration of the experiment (Beckage, Lavine, & Clark, 2005; Connell & Green, 2000; Wright, Muller‐Landau, Calderón, & Hernandéz, 2005), and there is a lack of information from long‐term monitoring. If a large shift of CNDD occurs across years, it may have no consistent effects on population growth, making seedling dynamics appear loosely coupled with population dynamics and diversity (Zhu, Comita, Hubbell, & Ma, 2015; Zhu, Woodall, et al., 2015). Moreover, different life stages have their own generation characteristics; for example, the structure at a later stage can be offset and reflect early productivity and recruitment rate. If CNDD at the seedling stage persists during a long period, the degree of conspecific aggregation will decline at later life stages, due to lower survival of individuals growing in conspecific high‐density patches (Barot, Gignoux, & Menaut, 1999; Condit et al., 2000). That is, there are many species showing dispersed patterns of saplings and juveniles relative to adults. CNDD will also be detected at later life stages in tree communities (Zhu, Mi, Ren, & Ma, 2010). Therefore, long‐term monitoring is necessary to better understand CNDD at the community level and to explain ecological processes causing establishment patterns observed at later life stages.

In this paper, to assess the relationship between seedling dynamics and community structure, three complementary methods were used to quantify seedling density, seedling survival and the resulting spatial patterns of saplings and juveniles. We used data from 150 stations across a 25‐ha temperate forest plot in Northeast China, spanning 11 years to address the following three questions: (1) Are seedling density and survival related to local neighborhood variables including conspecific and heterospecific densities, as well as abiotic variables? (2) Is the relationship between seedling survival and conspecific density consistent with CNDD, and does this relationship change with seedling abundance? (3) Are spatial patterns of saplings and juveniles related to adult patterns for individual species in accordance with predictions of the CNDD at the seedling stage?

## 2 MATERIALS AND METHODS

### 2.1 Study site and data collection

This study was conducted in a 25‐ha Changbaishan (CBS) temperate forest dynamics plot in Northeastern China (Hao, Zhang, Song, Ye, & Li, 2007). All free‐standing individuals in the plot at least 1 cm in diameter at breast height (dbh; 1.3 m above ground) were tagged, measured and identified to the species level, and their geographic coordinates were recorded. The CBS plot census was carried out three times in 2004, 2009 and 2014. In this study, each individual in each census was assigned to one of five size classes (Tables 1 and S1). To monitor seed rain and seedling dynamics, 150 stations were set up in the plot. Each station consisted of one 0.5 m^2^ seed trap and three 1 m^2^ seedling plots placed 2 m away from three sides (west, north, and east) of the trap. Seed were collected twice a month from May to December and once per month from January to April between 2006 and 2014 (Wang et al., 2016). All seedlings <1 cm dbh in the seedling plot were counted, tagged, and identified in each seedling plot once (early summer) per year from 2005 to 2015. Recruited seedlings (tree seedlings of 1 year) every year were added and their status (alive/dead) was also tagged in the seedling census of the following year.

**Table 1:** Size classes, plant measurements made, and sampling methods for individual plants in the forest dynamics plot in an old‐growth temperate forest in Northeast China

Size class	Physical measurement	Sample method
Small seedling	Height ≤ 30 cm	Sampled in 1‐m^2^ plots
Large seedling	Height > 30 cm and dbh < 1 cm	Sampled in 1‐m^2^ plots
Sapling	1 cm ≤ dbh ≤ 10 cm	Mapped across forest
Juvenile	10 cm < dbh ≤ 20 cm	Mapped across forest
Adult	dbh > 20 cm	Mapped across forest

A different classification was used for two small tree species (*Prunus padus* and *Acer tegmentosum*, dbh < 30 cm): sapling, 1 cm ≤ dbh ≤ 6.25 cm; juvenile, 6.25 cm < dbh ≤ 12.5 cm; adult, dbh > 12.5 cm.

### 2.2 Biotic and abiotic variables

Biotic variables were quantified using four local neighborhood density parameters. Seedling neighbors, conspecific and heterospecific neighborhood seedling densities (hereafter Ncon and Nhet) in each year were calculated as the number of conspecific and heterospecific seedling neighbors within the 1 m^2^ seedling plot where the focal seedling was located. For individuals ≥1 cm dbh, neighbors, as well as conspecific and heterospecific densities (hereafter Acon and Ahet) were calculated by summing the basal area of conspecific and heterospecific individuals ≥1 cm dbh within a 20 m radius divided by the distance between each individual and the center of the seedling plot. To get a more precise neighborhood basal area index over the 11‐year period, the tree individual dbh data were retrieved from the plot census closest to the year of the focal seedling census. A 20‐m radius was chosen because tree species interactions are minimal beyond 20 m (Wang et al., 2010) and a 20‐m radius had been predicted to yield a better fit for seedlings dynamics (Bai et al., 2012).

Abiotic factors during this period were represented by topographic variables, which can influence moisture, solar radiation conditions, and dispersal. Three topographic variables were identified: elevation, convexity, and slope, which provided indirect measures of light and water availability. Elevation was measured at the four corners of a 20 × 20 m grid in the 25‐ha plot. We then calculated the elevation in a 1 × 1 m grid using kriging interpolating methods. Elevation was defined as the elevation of the center point of the seedling plot. Convexity and slope values were calculated for each seedling plot. Slope was defined as the single average angle from the horizontal of the entire quadrat. All topographic variables were calculated in the statistical software R version 3.3.1 (R Development Core Team, 2014). The kriging interpolating method used the geoR package (Ribeiro & Diggle, 2001). There were limited correlations among elevation, convexity, and slope in the 450 seedling plots (Table S3).

### 2.3 Model evaluation

Generalized linear mixed models (GLMMs) were used for analyzing seedling dynamics over multiple years because census year can be included as a random effect quantifying interannual variation (Liu et al., 2012; Metz, Sousa, & Valencia, 2010). We also included species as a random effect in the community‐level models, since baseline species densities and survival rates varied widely (Chen et al., 2010). In addition, seedling plot was included as a random effect to take into account spatial autocorrelation (Bai et al., 2012).

In order to test the relative importance of biotic variables and abiotic variables for seedling dynamics, all models for density and survival were evaluated using the following four candidate models: (1) a null model including only census year, species and seedling plot as random effects, (2) a biotic model in which local neighborhood density was added to the null model, (3) an abiotic model in which the fixed effects of topography were added to the null model, and (4) a full model in which the fixed effects of all biotic and abiotic variables were added to the null model (Table S2). All models were compared using Akaike's Information Criterion (AIC). When comparing models with AIC difference <2, models were judged as equally valid (Burnham & Anderson, 2003). Values for all explanatory variables in all models were standardized prior to running the model by subtracting the mean of the variable and dividing by the standard deviation. To avoid boundary effects, we excluded those seedlings with a distance less than or equal to 20 m from the 25‐ha plot edges. In total, 126 seedling plots were included in our analyzes.

### 2.4 Data analysis

In order to better understand forest regeneration dynamics, three types of analyzes were respectively used to test the patterns of density and survival of tree seedlings and the spatial patterns of saplings and juveniles.

First, we used GLMMs with a negative binomial error to test whether seedling (recruited seedling and live seedling) density was related to local neighborhood density and topographic variables described above. Local neighborhood density included Acon and Ahet. For abiotic factors, we included elevation, convexity and slope. Recruited seedling density was the number of recruited seedlings in the seedling plot in each census year. Live seedling density was the sum of recruited seedlings and surviving seedlings of the previous seedling census. The analyzes were conducted at three levels: tree community level analyzes (all tree species combined in the whole dataset), individual species level analyzes (those that occurred in >40 of the seedling plots) and a time series analyzes (census year). To evaluate interannual variation of the relationship between conspecific density and seedling density across years we used the coefficient of variation (CV_year_), the standard deviation of the estimate of Acon divided by the mean (Mean_year_).

Second, we used GLMMs with binomial errors to model seedling (recruited seedling and live seedling) survival over 1 year as a function of local neighborhood density and topographic variables. Besides Acon and Ahet, conspecific and heterospecific seedling density (Ncon and Nhet) were also included as neighborhood density in the seedling survival models. To better illustrate the general pattern of seedling survival over 10 years, the analyzes were also conducted at four levels: tree community level analyzes (all tree species combined in the whole dataset), individual species level analyzes (numbers >40), a time series analyzes and a height class level (only for live seedling).

Third, we used spatial pattern analyzes based on a bivariate pair correlation function to estimate conspecific density‐dependent thinning from the sapling and juvenile to adult stage in three plot censuses. To account for species with specific habitat associations, the null model was a heterogeneous Poisson process with an Epanechnikov kernel for density estimation with a bandwidth of 30 m and a spatial resolution of 1 m. For all spatial point pattern analysis, we performed 99 Monte Carlo simulations and used the bivariate pair correlation function g_12_(r) as a test statistic to test for significant departures from the null model. In order to know how saplings and juveniles were distributed within local neighborhoods of adults, we left adults untouched (pattern 1) and distributed saplings and juveniles (pattern 2) using the heterogeneous Poisson process. The fifth‐lowest and fifth‐highest values were treated as simulation envelopes. Values inside the confidence interval support the null model, indicating a random spatial pattern of saplings or juveniles relative to trees. Observed densities less than the 99% confidence intervals indicate a dispersed spatial pattern, while densities greater than the 99% confidence intervals indicate a clustered spatial pattern.

In addition, we selected *Fraxinus mandshurica* and *Tilia amurensis* to examine the relation between the validity of CNDD and seedling abundance. These two species both had significant CNDD and generally had high seedling abundance in the CBS plot. For each species, we randomly chose a small subset of seedling and examined the significance of CNDD using GLMMs with a negative binomial error. The random choice was performed 1,000 times. The rate of the time with significant CNDD was calculated as the validity of the focal seedling abundance. With seedling abundance increased, the validity of CNDD was calculated at the different seedling abundance.

All analyzes were carried out in the statistical software R version 3.3.1 (R Development Core Team, 2014), using the ‘lme4’ package (Bates, Mächler, Bolker, & Walker, 2015) for GLMMs and ‘spatstat’ package (Baddeley, Rubak, & Turner, 2015) for spatial pattern analyzes.

## 3 RESULTS

### 3.1 Seedling density pattern

**Figure 1:**
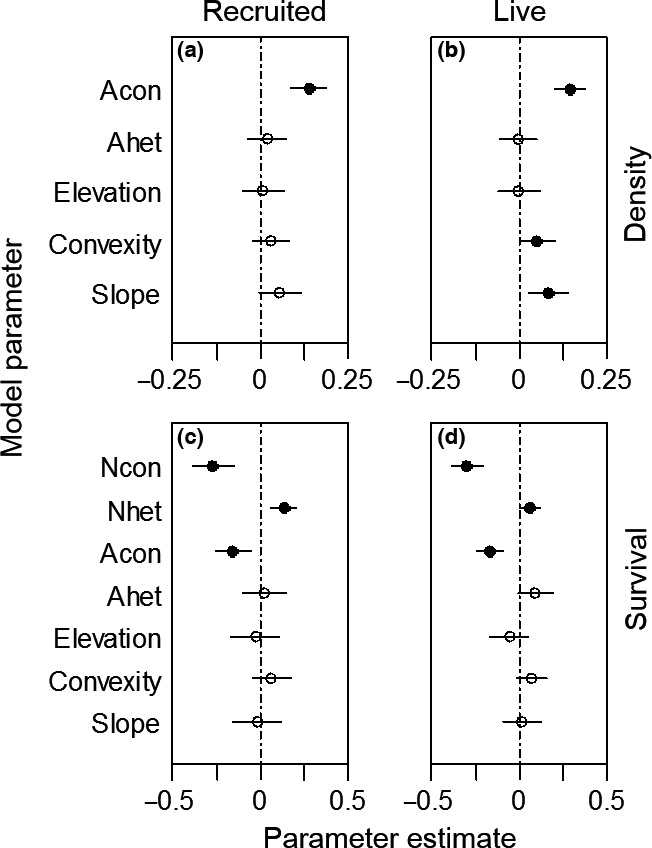
Parameter coefficient estimates (±2 *SE*) of density (first row) and survival (second row) of recruited and live seedlings at the community level over 11 years. Solid symbols indicate parameters significantly different than zero (*p* < .05)

**Figure 2:**
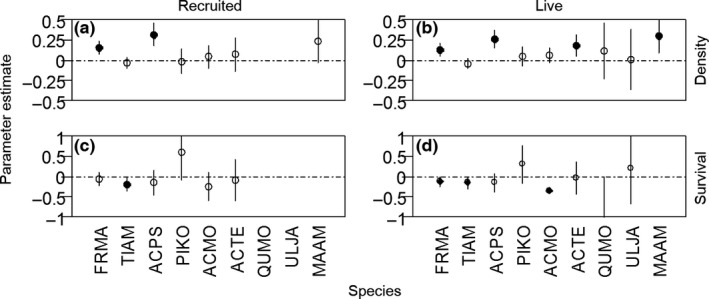
Parameter coefficient estimates of conspecific neighborhood basal area index (Acon) from generalized linear models of seedling density and survival for nine dominant species. Note species abbreviations: FRMA,* Fraxinus mandshurica*; TIAM,* Tilia amurensis*; ACPS,* Acer pseudo‐sieboldianum*; PIKO,* Pinus koraiensis*; ACMO,* Acer mono*; ACTE,* Acer tegmentosum*; QUMO,* Quercus mongolica*; ULJA,* Ulmus japonica*; MAAM,* Maackia amurensis*. Data is missing, because the number of seedling plot or seedling abundance is low than 40

**Figure 3:**
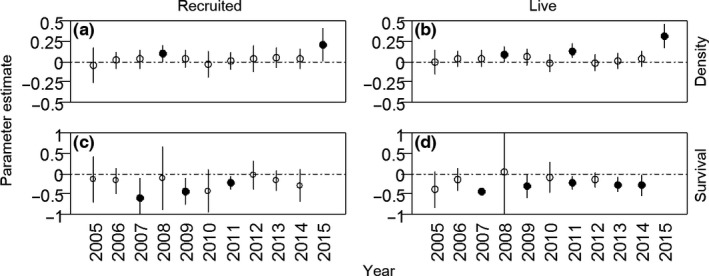
Parameter coefficient estimates of conspecific neighborhood basal area index from generalized linear models of seedling density and survival for data collected each year

For recruited seedlings, the biotic model had the lowest AIC. The full model was not significantly different from the biotic model. None of the abiotic variables had significant effects. For live seedlings, the full model was the best model (Table 2). The density of all seedlings was significantly positively correlated with Acon in the most likely models (recruited seedlings, Figure 1a; live seedlings, Figure 1b). At the individual species level, 2 of 6 species (33.3%) of the recruited seedling had significantly positive correlations (Figure 2a), while 4 of 9 species (44.4%) of recruited seedling did (Figure 2b). Although topographic variables had no significant effects on recruited seedlings, convexity and slope had significant positive effects on live seedlings (Figure 1a), largely caused by *F. mandshurica* and *Acer pseudo‐sieboldianum* (Fig. S1). Acon had a positive effect on seedling density in most of the 11 census years, of which 2008, 2011 and 2015 were significant, but strength varied across years for recruited seedlings (Mean_year_ = 0.041, CV_year_ = 1.686) and live seedlings (Mean_year_ = 0.061, CV_year_ = 1.568) (Figure 3a,b).

**Table 2:** AIC and ΔAIC values derived from density and survival models for recruited and live seedlings

Instances	Level	Type	Models							
			Null		Biotic		Abiotic		Full	
			AIC	△AIC	AIC	△AIC	AIC	△AIC	AIC	△AIC
Density										
	Community									
		Recruited seedlings	15,643.16	34.08	15,609.08	**0**	15,643.56	34.48	15,610.70	**1.62**
		Live seedlings	22,933.58	73.64	22,865.98	6.04	22,924.89	64.95	22,859.94	**0**
Survival										
	Community									
		Recruited seedlings	10,650.64	71.96	10,578.68	**0**	10,654.28	75.60	10,583.04	4.36
		Live seedlings	16,455.60	116.87	16,338.73	**0**	16,458.92	120.19	16,341.18	2.45
	Height									
		<30 cm	15,940.05	111.50	15,828.55	**0**	15,943.26	114.71	15,831.88	3.33
		>30 cm	274.37	**0**	278.06	3.69	279.91	5.54	283.54	9.17

Values in bold indicate ΔAIC values <2.

### 3.2 Seedling survival pattern

The biotic model provided the best fit for seedling survival when controlling for species, census year, and spatial location (Table 2). There were qualitatively similar results between biotic and abiotic variables for recruited seedlings (Figure 1c) and live seedlings (Figure 1d). Acon had a significant negative effect on seedling survival at the community level (Figure 1c,d), and for 3 of 8 (37.5%) individual species (Figure 2c,d). In contrast, Ncon was positively correlated with seedling survival at the community‐level (Figure 1c,d), but *F. mandshurica* showed a significant negative relationship between seedling survival and Ncon, *A. mono* and *A*. *pseudo‐sieboldianum* had significant positive correlations at the individual species level (Fig. S2). Nhet had a significant positive effect on seedling survival at the community level (Figure 1c,d), while at the individual species level, the effects of Nhet were not significant, only one (*F. mandshurica*) of eight species (Fig. S2). Abiotic variables were not significantly correlated with seedling survival (Figure 1c,d). For the time series analyzes, 3 of 10 years (30%) showed significant negative correlations between seedling survival and Acon for recruited seedlings (Figure 3c), while in 5 of 10 years (50%), they were negatively correlated with live seedlings (Figure 3d). Analyzes for different height classes for live seedlings over 10 years indicated that the biotic model was the most likely model for small seedlings, but the null model provided the best‐fit for large seedlings (Table 2, Fig. S3).

### 3.3 Spatial pattern analyzes of saplings and juveniles

**Figure 4:**
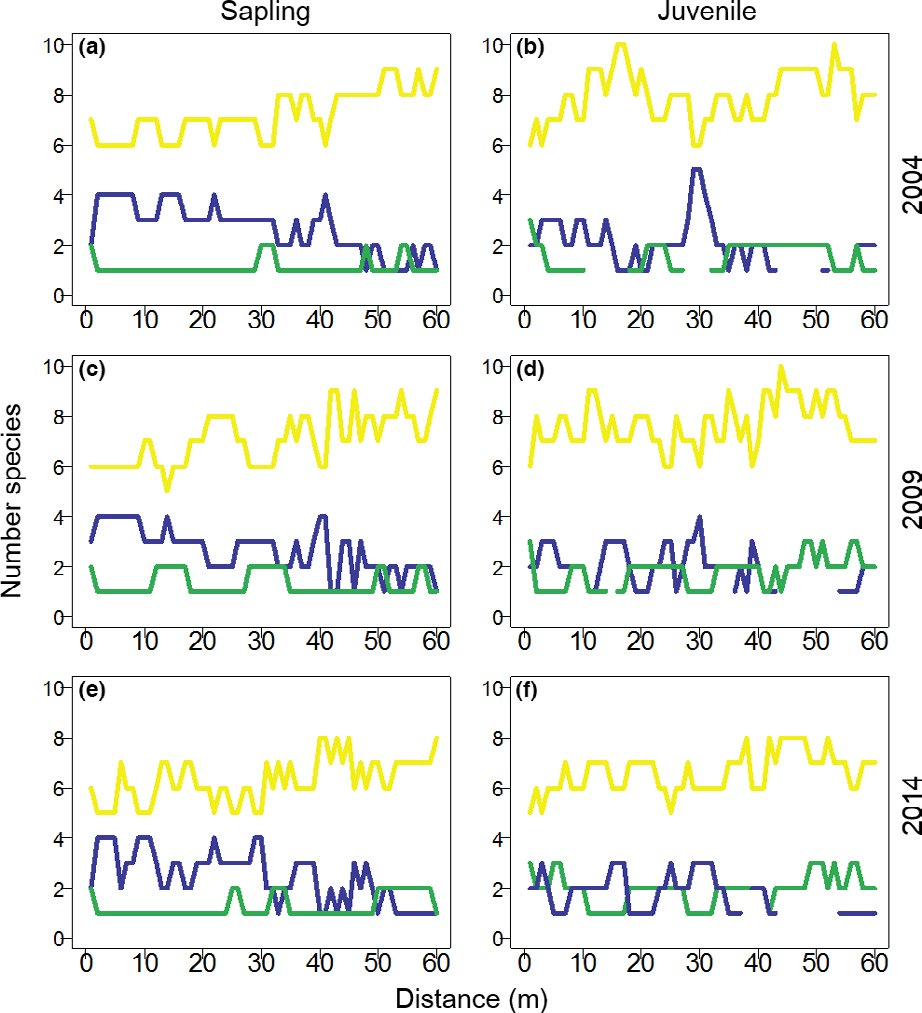
Spatial point pattern analysis using bivariate O‐ring statistic for sapling and juvenile relative to conspecific adults in three plot censuses (2004, 2009 and 2014). Data represent the number of species that exhibited a dispersed (blue), clustered (green), or random (yellow) point pattern at discrete distance rings from their conspecific adult

The number of species showing clustered, random, and dispersed patterns of saplings and juveniles relative to adults was calculated at each (1 m) scale up to 60 m. For saplings, 4 of 11 species (36.4%) exhibited a dispersed pattern. Only one species showed a clustered pattern at a scale up to 10 m while most species showed a significant random distribution pattern (Figure 4a,c,e). A large number of species that had a significant dispersion declined at >10 m scale (Figure 4a,c,e). Compared to the sapling stage, few species at the juvenile stage had a dispersed pattern (Figure 4b,d,f). There were similar spatial patterns both for saplings and juveniles during the three plot censuses spanning 10 years, suggesting that spatial pattern was relatively consistent in the temperate forest.

## 4 DISCUSSION

Seedlings are sensitive to many biotic and abiotic variables (Lu et al., 2015; Paine et al., 2011; Queenborough, Burslem, Garwood, & Valencia, 2007; Shibata et al., 2010) and their success and distribution can contribute to stabilizing community structure and diversity (Beckage et al., 2005; Connell & Green, 2000; Grubb, 1977). Using 11 years of data and three types of analyzes, we examined the effects of conspecific dependence on community structure while simultaneously accounting for other biotic and abiotic factors. Our results show that conspecific density impacts seedling dynamics, which in turn determines community structure. In particular, areas with a high conspecific density generally had high seedling density due to dispersal limitation. Seedling survival declined sharply with increasing conspecific density caused by conspecific negative density dependence allowing other species to be recruited and slowing intercompetitive exclusion. Although there were interspecific and interannual variations of CNDD on seedling survival, its effect was consistent across years and resulted in a dispersed pattern of saplings relative to adults. Also, habitat preference was found to affect seedling dynamics. Overall, dispersal limitation, habitat preference and conspecific negative density dependence affect seedling dynamics, leading to the distribution pattern observed at later life stages.

### 4.1 Dispersal limitation, habitat preference and conspecific negative density dependence in seedling dynamics

Dispersal limitation, habitat preference and CNDD are important mechanisms driving tree seedling assemblages. Seed arrival (dispersal limitation) and habitat preference have been previously shown to explain the abundance of woody species at the seedling stage in tropical old‐growth forests (Makana & Thomas, 2004; Norden et al., 2009). In this study, there was a strong significant positive correlation between seed density and Acon in the CBS plot (Fig. S4). Additionally, we found that conspecific density was included in all the best fitting models of seedling density. Density of seeds, recruited and live seedlings at the community level was higher where conspecific density was higher, suggesting that dispersal limitation is an important factor in determining tree seedling populations in temperate forests. The finding that dispersal limitation may drive seedling dynamics was also found in three large mapped forest plots in Indiana, Virginia, and Wisconsin, USA (Johnson et al., 2014). Live seedling density correlated with habitat variables such as convexity and slope, which may indirectly reflect that tree seedling species favor special light or water conditions. Live seedlings included recruited seedlings (1‐year‐old seedlings) and seedlings older than 1 year. The significant correlation between live seedling density and habitat conditions indicates that habitat preference also contributes to seedling establishment particularly at older seedling ages.

Seedlings readily occurred with a high conspecific density as a result of dispersal limitation, but the survival of these seedlings decreased with increasing conspecific density. That is, CNDD did occur in the CBS plot. The strength of the effects of Acon density showed a decline from seed, recruited to live seeding stages during the 11‐year period, indirectly indicating that CNDD exists in the seed‐to‐seedling transition. CNDD has been found in other temperate forest sites, as well as tropical and subtropical forests (Chen et al., 2010; Metz et al., 2010; Piao, Comita, Jin, & Kim, 2013), but these conclusions come largely from short‐term studies. CNDD at the community level was found over the 11‐year study period in the current study. For local neighborhood individuals, individual performance was limited more by conspecific density than heterospecific density (Johnson et al., 2012; Volkov, Banavar, He, Hubbell, & Maritan, 2005). We also found that the effect of heterospecific density on seedling survival was generally not significant compared to conspecific density. For the effects of seedling neighbors on seedling survival, conspecific and heterospecific seedling density both had positive contributions for most species potentially because seedling survival of all species is higher in optimal habitats (Johnson et al., 2014). Previous studies have shown that seedling–seedling competition is weak in tropical forests compared to the influence of habitat (Terborgh, 2012; Timothy Paine, Harms, Schnitzer, & Carson, 2008). Nonetheless, there was a negative relationship between conspecific density and seedling survival at the community level, which was caused mainly by intraspecific competition for *F. mandshurica* (63.5% of all recorded tree seedlings). Owing to the negative correlation and high abundance of *F. mandshurica*, conspecific density showed a negative correlation with seedling survival.

### 4.2 Interspecific and interannual variations of conspecific negative density dependence

Conspecific negative density dependence (CNDD) is a widespread mechanism in the maintenance of diversity through regulation of conspecific population dynamics (Bever, Mangan, & Alexander, 2015; Comita et al., 2014; Hyatt et al., 2003). CNDD at the seedling stage is stronger than at other later life stages (Zhu, Comita, et al., 2015). We found a large variation of CNDD at the seedling stage among species and across years. At the individual species level, we found 37.5% of the species showing CNDD, which is similar to the number of species (1/3) showing CNDD in eastern North America (Johnson et al., 2014). Furthermore, CNDD was observed in five of the 10 years of the current study. The variation among species and across years is related to seedling abundance (Zhu, Woodall, et al., 2015). In our study, species and years with high seedling abundance generally showed significant CNDD. A study of tropical forests supports the theory that when conspecific individuals are abundant, soil pathogens suppress seedling recruitment, but little effect is observed when conspecific individuals are rare due to a lack of pathogens (Liu et al., 2015). Therefore, seedlings with low abundance are not uniformly distributed in different conspecific density gradients. That is, seedlings tend to cluster where there is a high conspecific or low density, making it difficult to produce an effective CNDD. Previous studies disagree about whether CNDD is greatest for rare species (Johnson et al., 2012; Klironomos, 2002; Mangan et al., 2010), or for common species (Bagchi et al., 2014; Kobe & Vriesendorp, 2011; Webb & Peart, 2000; Zhu, Woodall, et al., 2015). Such discrepancies likely result from different species and seedling abundances. Small sample size and low seedling abundance result in rare species infrequently cooccurring with conspecific neighbors, which make it difficult to identify significant relationships (Zhu, Woodall, et al., 2015). Common species sometimes have low seedling abundance, while the low seedling abundance could affect the effective impacts of CNDD. For example, CNDD was extremely significant for *F. mandshurica* and *T. amurensis* (Figure 2d), but the validity of CNDD was reduced when seedling abundance was low (Fig. S5). Although common species are expected to have a stronger CNDD compared to rare species, CNDD often has a low validity when common species have a low seedling abundance. Owing to the variation of seedling abundance among species and across years, adequate seedling abundance in any study is necessary for CNDD detection.

### 4.3 Spatial patterns of saplings and juveniles

Although the dynamics at early life stages may determine the community structure at later life stages, seedling survival patterns loosely correlate with spatial patterns of saplings and juveniles due to interspecific and interannual variations in seedling production and mortality (Cook, 1979; Wright et al., 2005). A consistent temporal trend in seedling dynamics can contribute to a stable community structure, and determine the successional trajectory of forests.

The results of the current study show that although spatial patterns of the species examined varied, approximately 40% species exhibited a dispersed sapling distribution relative to conspecific adults from 0 to 10 m. This suggests that the persistence of CNDD at the seedling stage has an important effect on distribution patterns at later life stages. Eleven of the 15 focal species have previously shown conspecific density‐dependent thinning in a similar temperate forest also in Northeast China (Piao et al., 2013). The dispersed pattern of saplings relative to conspecific adults may result from conspecific negative density dependent mortality at the seedling stage. This diminishes conspecific neighbor density, such that large trees are surrounded by relatively few conspecific trees (Zhu, Comita, et al., 2015). The number of species with a significantly dispersed sapling distribution decreases sharply at a scale distance >10 m, potentially due to distance‐dependent seed and seedling mortality (Comita et al., 2014). In the Gutianshan subtropical forest plot, the aggregation pattern of saplings peaks at 5 m from adults (Zhu, Getzin, Wiegand, Ren, & Ma, 2013). This indirectly verifies that distance dependent seedling mortality is happening near adults and influences the resulting spatial association of saplings and adults. Unfortunately there is no seedling analysis to directly support this theory. However, the spatial distribution of juveniles relative to conspecific individuals was not significantly different. The number of species with a dispersed distribution pattern was almost the same as that of species with clustered distribution patterns. This is likely caused by a decrease in the strength of conspecific negative density dependence as individuals aged (Comita & Hubbell, 2009). Zhu, Woodall, et al., 2015 found that adult tree survival was positively correlated with conspecific density, indicating that the impact of conspecific density at later life stages may be negligible compared to other factors such as interspecific competition and/or environmental conditions. The spatial distribution pattern of saplings and juveniles was relatively consistent during 10 years, indicating in turn that CNDD at the seedling stage is a long‐term effect.

### 4.4 Caveats

Our study presents comprehensive analyzes of conspecific density dependence over an 11‐year period. Nevertheless, one major limitation of our study is that climatic change is not included. CNDD is predicted to be stronger in wetter habitats than drier habitats (Comita et al., 2014). In warmer and wetter years, common species have higher CNDD than rare species. The variation in CNDD on seedling survival of common and rare species could have important consequences for the maintenance of tree species diversity (Bachelot, Kobe, & Vriesendorp, 2015). Attention must be paid to climate change in future work.

## 5 CONCLUSION

Over 11 years of monitoring in a temperate forest, we found that conspecific density had a positive effect on seedling abundance, but a negative effect on seedling survival. Spatial analysis suggests that more species exhibit a dispersed sapling distribution relative to conspecific adults compared to species with a clustered pattern at small scales. Overall, we show that conspecific density dependence influences community structure at later life stages.

## CONFLICT OF INTEREST

We declare that there is no conflict of interest.
