# Supplementary material for: Conspecific density dependence and community structure: Insights from 11 years of monitoring in an old‐growth temperate forest in Northeast China
Source: Ecol Evol. 2017 Jun 7;7(14):5191–200. doi: 10.1002/ece3.3050 (PMC5528227; doi:10.1002/ece3.3050)
Supplement: Supplementary file 1 [file ECE3-7-5191-s001.doc]

**Supplementary information**

Table S1. Information of main species in the plot censuses of 2004, 2009 and 2014.

| Species | Maximum dbh (cm) | Abundance in 2004 | | | Abundance in 2009 | | | Abundance in 2014 | | |
| --- | --- | --- | --- | --- | --- | --- | --- | --- | --- | --- |
|  |  | Sapling | Juvenile | Adult | Sapling | Juvenile | Adult | Sapling | Juvenile | Adult |
| *Acer mono* | 61 | 4905 | 870 | 794 | 4840 | 864 | 775 | 5240 | 803 | 736 |
| *Acer pseudo-sieboldianum* | 48.6 | 3928 | 904 | 59 | 3655 | 972 | 63 | 4328 | 401 | 32 |
| *Tilia amurensis* | 104.4 | 438 | 299 | 1907 | 324 | 301 | 1879 | 340 | 255 | 1761 |
| *Pinus koraiensis* | 98.5 | 26 | 446 | 1978 | 19 | 385 | 1947 | 10 | 335 | 1917 |
| *Ulmus japonica* | 100.1 | 696 | 156 | 249 | 641 | 174 | 250 | 686 | 172 | 250 |
| *Quercus mongolica* | 104.2 | 147 | 54 | 725 | 142 | 53 | 710 | 135 | 51 | 699 |
| *Maackia amurensis* | 35.4 | 385 | 323 | 40 | 464 | 153 | 70 | 521 | 143 | 29 |
| *Fraxinus mandshurica* | 100.3 | 39 | 67 | 588 | 24 | 66 | 577 | 26 | 54 | 575 |
| *Acer tegmentosum* | 23 | 473 | 152 | 62 | 275 | 324 | 43 | 185 | 266 | 42 |
| *Prunus padus* | 27.2 | 364 | 80 | 41 | 325 | 82 | 45 | 345 | 74 | 32 |
| *Tilia mandshurica* | 76.5 | 274 | 79 | 69 | 192 | 73 | 71 | 152 | 59 | 70 |
| *Acer triflorum* | 41.5 | 173 | 78 | 27 | 165 | 73 | 29 | 172 | 76 | 28 |
| *Acer mandshuricum* | 47.2 | 175 | 43 | 18 | 169 | 43 | 21 | 175 | 45 | 21 |
| *Ulmus laciniata* | 56 | 143 | 16 | 12 | 137 | 18 | 12 | 163 | 23 | 11 |
| *Malus baccata* | 48.3 | 98 | 22 | 30 | 101 | 25 | 26 | 131 | 27 | 25 |

Note: Maximum dbh represents the maximum dbh of each species in three plot censuses.

Table S2. Model structures for analyzing seedling density and survival.

| Model class |  | Model structure |
| --- | --- | --- |
| Null model |  | y ~ (1|year) + (1|species) + (1|plot) |
| Biotic model |  | y ~ a + b_1_ × Bio_1_ + b_2_ × Bio_2_ + ... b_n_ × Bio_n_  + (1|year) + (1|species) + (1|plot) |
| Abiotic model |  | y ~ a + c_1_ × Abio_1_ + c_2_ × Abio_2_ + ... c_n_ × Abio_n_ + (1|year) + (1|species) + (1|plot) |
| All model |  | y ~ a + b_1_ × Bio_1_ + b_2_ × Bio_2_ + ... b_n_ × Bio_n_  + c_1_ × Abio_1_ + c_2_ × Abio_2_ + ... c_n_ × Abio_n_ + (1|year) + (1|species) + (1|plot) |

Note: Response variable y represents seedling density for seedling density model, and seedling survival for seedling survival model. Bio_1_ - Bio_n_ are biotical variables. Abio_1_ - Abio_n_ are abiotical variables. b_1_ - b_n_ and c_1_ - c_n_ are model coefficients. a is model intercept. Seedling census year (year), species, seedling plot (plot) are random effects.

Table S3. Correlations among elevation, convexity, and slope in the 450 seedling plots.

|  | Correlation coefficient | P |
| --- | --- | --- |
| Elevation and slope | -0.538 | < 0.01 |
| Elevation and convexity | 0.278 | < 0.01 |
| Slope and convexity | -0.301 | < 0.01 |

Figure S1. Parameter coefficient estimates of convexity and slope from generalized linear models of live seedling density for nine dominant species.

Figure S2. Parameter coefficient estimates of conspecific neighborhood seedling density (Ncon) and hetrospecific neighborhood seedling density (Nhet) from generalized linear models of seedling survival for eight dominant species.

Figure S3. Parameter coefficient estimates of different height classes of live seedling survival over 10 years. Small seedling (Small): height ≤ 30 cm; Large seedling (Large): height > 30 cm and dbh < 1 cm.

Figure S4. The relations between seed density and biotic and abiotic variables. Seed density is defined as the seed number over two seedling census intervals. A: Parameter coefficient estimates of seed density at the community level over 11 years,. B: Parameter coefficient estimates of conspecific density from generalized linear models of seed density for twelve dominant species, C: Parameter coefficient estimates of conspecific density from generalized linear models of seed density for data collected each year. All analyses were based seed rain data of 126 seed traps from May 2006 to April 2014.

Figure S5. Probability of conspecific negative density dependence mortality with significance across seedling abundance for *Fraxinus mandshurica* and *Tilia amurensis.*


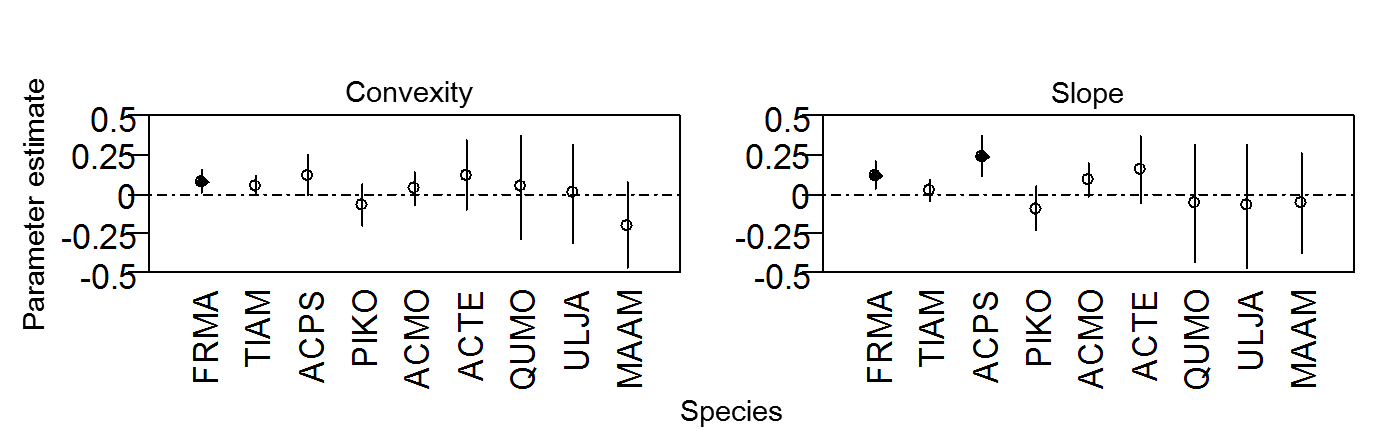


**Figure S1.**

**
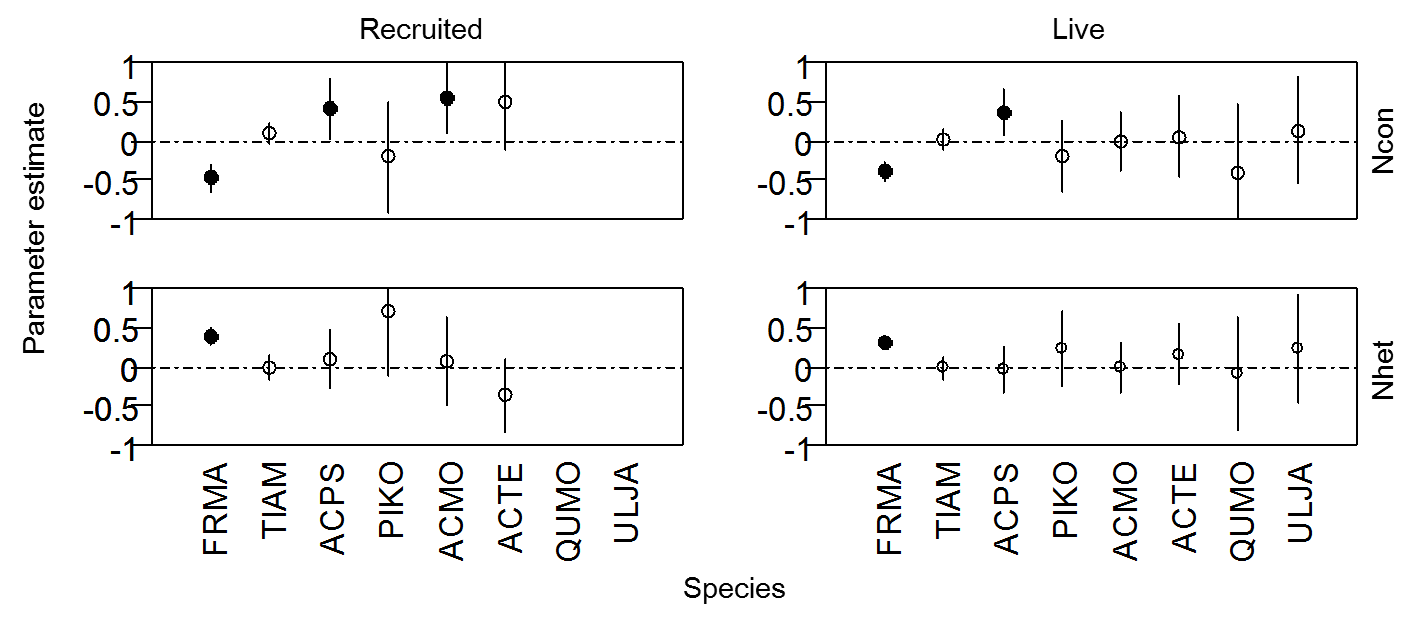
**

**Figure S2.**


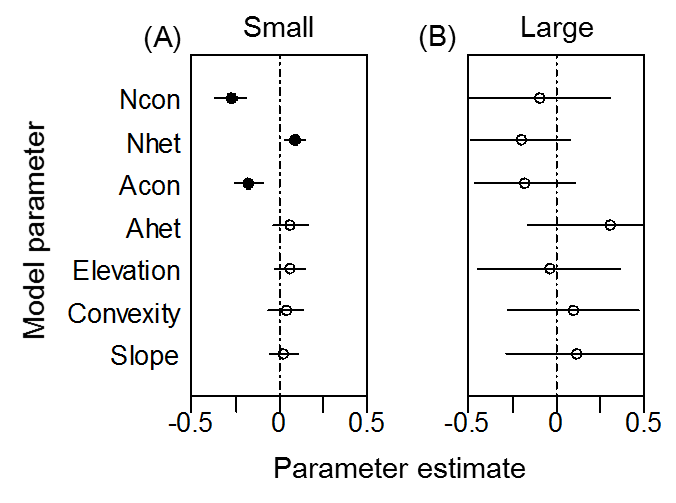


**Figure S3.**

**
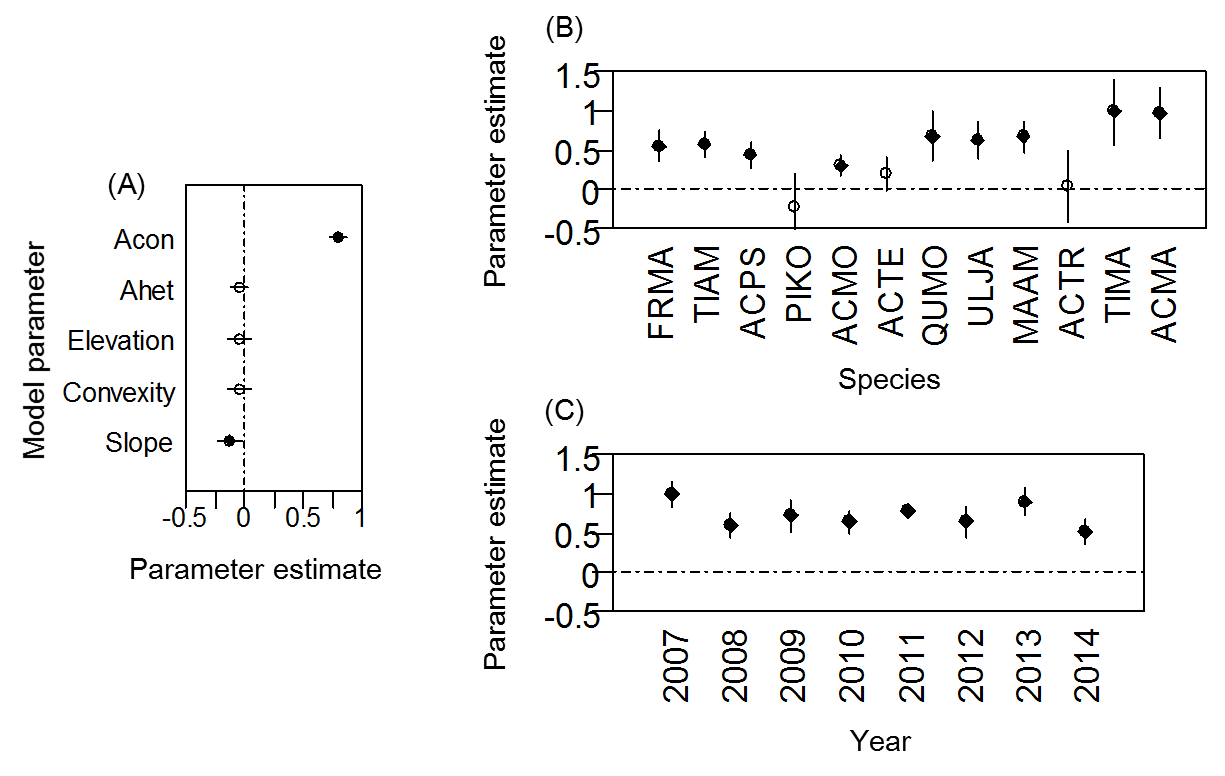
**

**Figrue S4.**

**
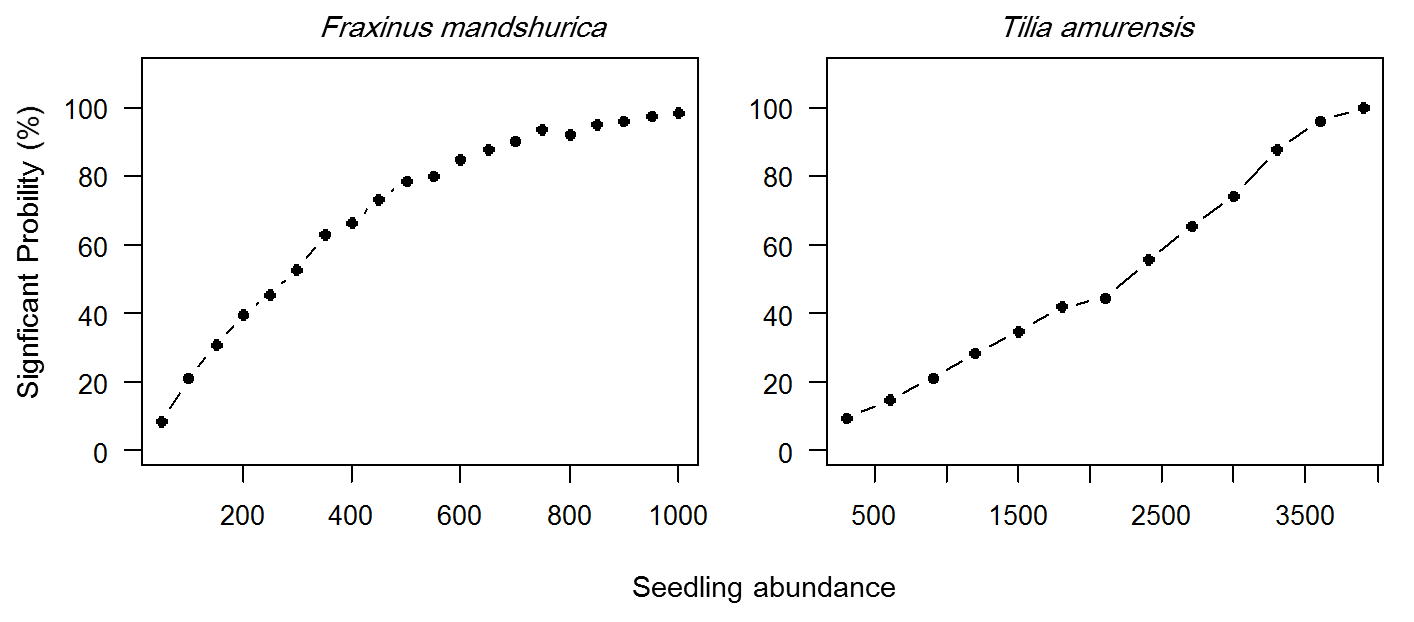
**

**Figure S5.**
